# Low barrier medication for opioid use disorder at a federally qualified health center: a retrospective cohort study

**DOI:** 10.1186/s13722-022-00342-1

**Published:** 2022-11-05

**Authors:** Jamie Carter, Zhen Li, Hillary Chen, Melissa Greiner, Christopher Bush, Debanjan Bhattacharya, Stephanie Poley, Nidhi Sachdeva, Jane Carolyn Crowder, Jacob Feigal

**Affiliations:** 1 Lincoln Community Health Center, 1301 Fayetteville St, Durham, NC 27707 North Carolina US; 2 Department of Population Health Sciences, Duke University School of Medicine, Durham, US; 3 Department of Psychiatry and Behavioral Sciences, Duke University School of Medicine, Durham, US; 4 Department of Medicine, Duke University School of Medicine, Durham, US

**Keywords:** Opioid use disorder, Medication for opioid use disorder, Buprenorphine, Harm reduction

## Abstract

**Background:**

Medication for opioid use disorder (MOUD) reduces mortality, but few patients access MOUD. At a Federally Qualified Health Center (FQHC), we implemented a low barrier model of MOUD, including same-day MOUD initiation and a harm reduction philosophy.

**Objective:**

To investigate whether low barrier MOUD improved retention in care compared to traditional treatment.

**Design and participants:**

Retrospective cohort study of patients with at least one visit seeking MOUD at the FQHC during a historical control period (3/1/2018—3/31/2019) and a low barrier intervention period (11/1/2019—7/31/2020).

**Main measures:**

Primary outcomes were any MOUD prescription within 6 months of the index visit and 3- and 6-month retention in treatment without care gap, with care gap defined as 60 consecutive days without a visit or prescription. Secondary outcomes were all-cause hospitalization and emergency department visit within 6 months of the index visit.

**Key results:**

Baseline characteristics were similar between the intervention (n = 113) and control (n = 90) groups, except the intervention group had higher rates of uninsured, public insurance and diabetes. Any MOUD prescription within 6 months of index visit was higher in the intervention group (97.3% vs 70%), with higher adjusted odds of MOUD prescription (OR = 4.01, 95% CI 2.08–7.71). Retention in care was similar between groups at 3 months (61.9% vs 60%, aOR = 1.06, 95% CI 0.78–1.44). At 6 months, a higher proportion of the intervention group was retained in care, but the difference was not statistically significant (53.1% vs 45.6%, aOR 1.27, 95% CI 0.93–1.73). There was no significant difference in adjusted odds of 6-month hospitalization or ED visit between groups.

**Conclusions:**

Low barrier MOUD engaged a higher risk population and did not result in any statistically significant difference in retention in care compared with a historical control. Future research should determine what interventions improve retention of patients engaged through low barrier care. Primary care clinics can implement low barrier treatment to make MOUD accessible to a broader population.

**Supplementary Information:**

The online version contains supplementary material available at 10.1186/s13722-022-00342-1.

## Background

While opioid overdose death rates continue to rise [1, 2], medication for opioid use disorder (MOUD) substantially reduces mortality [3] and improves health and wellbeing [4]. However, a minority of patients with opioid use disorder (OUD) are treated with medication [5, 6]. Traditional office-based buprenorphine treatment has often included multiple visits to initiate medication, requirements for abstinence, office-based inductions, mandatory counseling, and other processes that may function as barriers to care for some patients, particularly those with limited transportation, childcare, financial resources, and social support. Other barriers to MOUD initiation and retention include lack of treatment availability, cost, stigma against people who use drugs and use of MOUD, social isolation, discrimination, competing priorities, criminalization of substance use, and structural racism [7–14].

To improve patient engagement and retention on MOUD, treatment programs are increasingly implementing low barrier models of care. Although there is no universal definition of low barrier treatment, typical features of these programs include a harm reduction approach, same-day treatment initiation, flexible attendance policies, and non-traditional settings. Programs with a harm reduction approach prioritize the reduction of drug-related harms, improvements in health and quality of life, and reduction of overdose risk as the goals of treatment, taking a non-judgmental attitude in partnering with patients. [15, 16]

Low barrier methadone maintenance treatment was piloted in Europe and Canada and was successful in retaining marginalized patients in care, reducing mortality, and decreasing injection-related risk behaviors [17–21]. In the United States, an initial pilot study of low barrier buprenorphine through a harm reduction agency showed feasibility and comparable retention to traditional treatment [22]. Since then, low barrier buprenorphine has been expanded to different populations, including patients experiencing homelessness and with criminal legal system involvement, and to different settings, including mobile vans, syringe services programs, and telehealth [23–30]. These low barrier programs in non-traditional settings have been successful in engaging patient populations that have difficulty accessing traditional office-based treatment, while achieving lower retention rates than traditional treatment [23–25, 27]. More recently, regulatory changes and practical considerations related to the COVID-19 pandemic have led to many programs lowering barriers through telehealth [26–30], with comparable rates of retention, higher patient satisfaction, and increased access to buprenorphine [31]. Provision of buprenorphine through primary care clinics is critical for addressing the unmet need for treatment across the United States [32], and primary care clinics are increasingly exploring low barrier models of MOUD.

## Objective

In November 2019, our FQHC in North Carolina transitioned from a traditional office-based buprenorphine treatment model to a low barrier model of treatment, including same-day initiation of MOUD and a harm reduction approach. This study aims to determine whether treatment through the low barrier model was associated with improved rates of MOUD prescription and retention in care at 3 and 6 months compared to treatment through the traditional model.

## Methods

### Program description

The treatment setting is a large FQHC near downtown Durham, NC that treats a diverse population including the local community and patients from surrounding rural areas. The FQHC has provided buprenorphine treatment since 2016 and offered extended-release naltrexone (XR-NTX) for OUD from January 2019 to June 2020 through a state-funded grant. During the traditional office-based model, patients seeking treatment were scheduled for either a group information session and then a behavioral health assessment, or only a behavioral health assessment, prior to an initial visit with a medical provider to start MOUD.

In November 2019, we implemented low barrier treatment by eliminating the requirement for an assessment or information session prior to the medical visit. We established protected same-day MOUD visits on medical provider schedules for new appointments, such that any patient who walked in or was seen in the clinic for another reason could establish care on-demand. Patients who called requesting initial appointments could often be accommodated same-day or within a few days. The low barrier model centered harm reduction, with patients continuing to be treated who had ongoing opioid and other substance use. Patients were encouraged to consider higher levels of care and supportive services when appropriate and available, but access to MOUD was not conditional on this engagement.

In both models of care, patients were connected with one of several medical providers at the clinic who prescribe MOUD, and this provider typically became their primary care provider and addressed primary care needs during MOUD visits. All buprenorphine inductions were home or non-facility-based inductions. Patients had appointments weekly until their buprenorphine dose was stable and then followed up less frequently at intervals between every two weeks and 3 months depending on their clinical stability. All patients met briefly with both a behavioral health counselor and a medical provider at every visit, per clinic leadership preferences and to facilitate team-based care. Although patients had scheduled appointments, our behavioral health team could accommodate patients who needed flexibility and meet on a walk-in basis to facilitate consistent access to MOUD. During the COVID-19 pandemic, the clinic transitioned to mostly telehealth visits for several months and then resumed in-person visits with the option of telehealth if there were barriers to clinic attendance.

Uninsured patients typically paid a sliding scale fee of $10 for their appointments and could receive vouchers to cover the cost of buprenorphine at the FQHC’s on-site pharmacy. During low barrier implementation, grant funding covered the costs of the sliding scale fees for the first ten visits for patients on buprenorphine. For patients on XR-NTX, all visit and medication costs were paid by the state grant.

### Data sources

We extracted electronic health records (EHR) data from Duke’s Epic/Clarity database, including patient demographics, encounter dates, visit types, diagnosis and procedure codes, prescription order records, and death dates. The study was approved by the Duke Institutional Review Board.

### Study population

We included patients who had at least one office visit with an FQHC medical or behavioral health provider seeking MOUD, selecting the record with the earliest date the patient met with a member of the MOUD team as the patient’s index visit. We excluded patients with a buprenorphine or XR-NTX prescription from an FQHC provider in the 3 months preceding the index visit. We restricted the cohort to patients with index visits during: a) the low barrier intervention period (11/1/2019—7/31/2020) and b) the historical control period (3/1/2018—3/31/2019).

### Intervention and historical control groups

The intervention and historical control groups were defined by index visits within the low barrier intervention and historical control periods. We selected the historical control group to be as close in time to the intervention while allowing for 7 months of follow-up for outcome ascertainment without any overlap with the intervention period.

### Covariates

Information on age, gender, race, ethnicity, insurance status and tobacco use as of the index visit were extracted from the EHR. Gender was defined as male or female based on the patient’s gender identity listed in the EHR; there were no non-binary participants. Information on race was condensed into the categories of white and Black/other due to the Duke EHR cell suppression policy, which did not allow reporting of frequencies between one and ten nor data that could allow back calculation of those small cells. We searched EHR data between the index visit and 1 year prior for diagnosis of comorbid physical and mental health conditions, including diabetes, HCV infection, HIV infection, MRSA or MSSA infection, chronic pain, depression, schizophrenia spectrum or other psychotic disorder, and bipolar disorder based on validated coding algorithms (Additional file 1: Appendix: Table S1).

### Outcomes and measures

The primary outcomes were any MOUD prescription within 6 months of the index visit and 3- and 6-month retention in treatment without care gap [33–35]. Any MOUD prescription within 6 months was defined as any prescription of buprenorphine or XR-NTX within 6 months of index visit. Retention in treatment at 3 and 6 months without care gap was defined as an office or telemedicine visit with an MOUD medical or behavioral provider or MOUD prescription in the 3rd or 4th month following index visit (day 61–120 after index), or in the 6th or 7th month following index visit (day 151–210 after index), respectively, without a care gap. Care gap was defined as 60 consecutive days without an office/telemedicine visit or MOUD prescription.

Secondary outcomes were all-cause hospitalization and all-cause emergency department (ED) visit within 6 months of index visit. Hospitalization was defined by any inpatient claim including an ED visit that transferred to inpatient, while ED visit was defined as any ED encounter that did not result in an inpatient hospitalization. We included these utilization outcomes to provide information about rates of opioid-related harms and changes in health status.

Cells containing a value of 1 to 10 were suppressed in accordance with Duke EHR cell suppression policy.

### Statistical analysis

We summarized baseline patient characteristics and tested for differences by intervention status using chi-square tests for categorical variables and Wilcoxon rank sum tests for continuous variables.

We calculated percentages of 6-month MOUD prescription, 3- and 6-month retention without care gap, all-cause hospitalization, and all-cause ED visit, and tested for group differences using chi-square tests. We described the number of all-cause hospitalizations and ED visits within 6 months using means with standard deviations and medians with interquartile ranges and tested for differences by group using Wilcoxon rank sum tests. Logistic regression models were used to evaluate the multivariable adjusted associations between intervention group and primary and secondary outcomes. For multivariable modeling, we adjusted for age, sex, race, ethnicity, insurance status, and comorbid conditions listed in the baseline characteristics table (Additional file 1: Appendix: Table S1).

We descriptively calculated rates of MOUD prescriptions and office/telemedicine visits in each of the 1st through 6th months following index visit. We also descriptively calculated rates of 3- and 6-month retention in care without excluding those with a care gap as a secondary analysis to explore the extent to which treatment interruptions affected retention in care. We described the rates of 6-month all-cause mortality, determined by death dates in the EHR, and 6-month unintentional opioid overdose, based on validated coding algorithms (Additional file 1: Appendix: Table S2).

## Results

### Demographics

203 patients met study criteria, having at least one visit seeking MOUD at the FQHC, of which 90 had an index visit during the historical control period and 113 during the intervention period. All patients were prescribed buprenorphine except one patient in each of the intervention and historical control groups who received XR-NTX.

Baseline characteristics were mostly similar between the intervention and historical control groups (Table 1). The intervention group had higher rates of uninsured and public insurance, lower rates of private insurance, and higher prevalence of diabetes.

**Table 1:** Baseline demographic and clinical characteristics of historical control and intervention groups

Variable	Historical control group	Intervention group	p-value
N	90	113	
Demographics			
Age (years), Median (Q1, Q3)	41.0 (33.0, 50.0)	38.0 (30.0, 47.0)	0.15
Gender, Male	66 (73.3%)	76 (67.3%)	0.35
Race			0.64
White	41 (45.6%)	56 (49.6%)	
Black/other	49 (54.4%)	57 (50.4%)	
Ethnicity, Hispanic	^a^(1.1%–11.1%)	^a^(0.9%–8.8%)	0.43
Insurance			0.01
Uninsured	32 (35.6%)	47 (41.6%)	
Medicare/Medicaid	28 (31.1%)	48 (42.5%)	
Private	30 (33.3%)	18 (15.9%)	
Medical history			
Current smoker	64 (75.3%)	79 (71.2%)	0.52
Prior smoker	^a^(1.1%–11.1%)	^a^(0.9%–8.8%)	0.90
Clinical characteristics			
Diabetes	^a^(1.1%–11.1%)	14 (12.4%)	0.048
HCV infection [Sample et al.]	11 (12.2%)	17 (15.0%)	0.56
HIV infection[Cochran]	0 (0.0%)	^a^(0.9%–8.8%)	0.37
Staphylococcus aureus infection [MRSA or MSSA]	0 (0.0%)	^a^(0.9%–8.8%)	0.20
Chronic Pain	^a^(1.1%–11.1%)	^a^(0.9%–8.8%)	0.58
Depression	16 (17.8%)	18 (15.9%)	0.73
Schizophrenia spectrum and other psychotic disorders	^a^(1.1%–11.1%)	^a^(0.9%–8.8%)	0.49
Bipolar disorder	^a^(1.1%–11.1%)	^a^(0.9%–8.8%)	0.58
Inpatient visits and ED visits			
Number of all-cause hospitalizations in prior 12 months, Mean (SD), Median (Q1, Q3)	0.3 (0.7), 0.0 (0.0, 0.0)	0.4 (0.8), 0.0 (0.0, 0.0)	0.30
Number of ED visits in prior 12 months, Mean (SD), Median (Q1, Q3)	2.8 (4.2), 2.0 (0.0, 3.0)	3.2 (6.5), 1.0 (0.0, 3.0)	0.14

^*^Cells containing a value of 1 to 10 or additional cells that allow the back calculation of those small cells were suppressed in accordance with Duke EHR cell suppression policy. The range of percentages shown in parentheses indicate the possible range of values based on the suppressed cells

### Retention in care

Any MOUD prescription within 6 months of index visit was higher in the intervention group compared to the historical control group (97.3% vs 70%) (Table 2), with higher adjusted odds of MOUD prescription (aOR 4.01, 95% CI 2.08–7.71, p < 0.0001) in the multivariable model. Retention in care without a care gap was similar between the intervention and historical control groups at 3 months (61.9% vs 60%, aOR 1.06, 95% CI 0.78–1.44, p = 0.71). At 6 months, a higher proportion of patients in the intervention group were retained in care without a care gap, but the association was not statistically significant (53.1% vs 45.6%, aOR 1.27, 95% CI 0.93–1.73, p = 0.14) (Table 5).

**Table 2:** Rates of MOUD prescription, retention in care, and acute care utilization in historical control and intervention groups

Outcome	historical control group	intervention group	p-value
N	90	113	
6-month any MOUD prescription	63 (70.0%)	110 (97.3%)	< 0.001
3-month treatment retention without care gap	54 (60.0%)	70 (61.9%)	0.78
6-month treatment retention without care gap	41 (45.6%)	60 (53.1%)	0.29
6-month all-cause hospitalization	^a^(1.1–11.1%)	^a^(0.9–8.8%)	0.99
6-month emergency department utilization	40 (44.4%)	44 (38.9%)	0.43

^a^Cells containing a value of 1 to 10 or additional cells that allow the back calculation of those small cells were suppressed in accordance with Duke EHR cell suppression policy

**Fig. 1:**
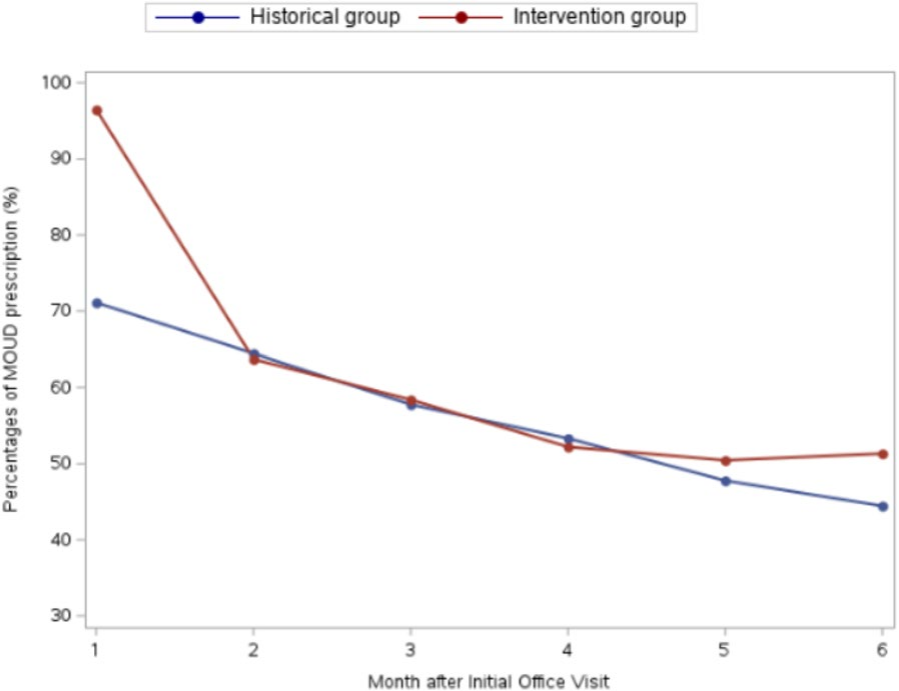
Percentage of patients with an MOUD prescription by month in intervention and historical control groups

**Fig. 2:**
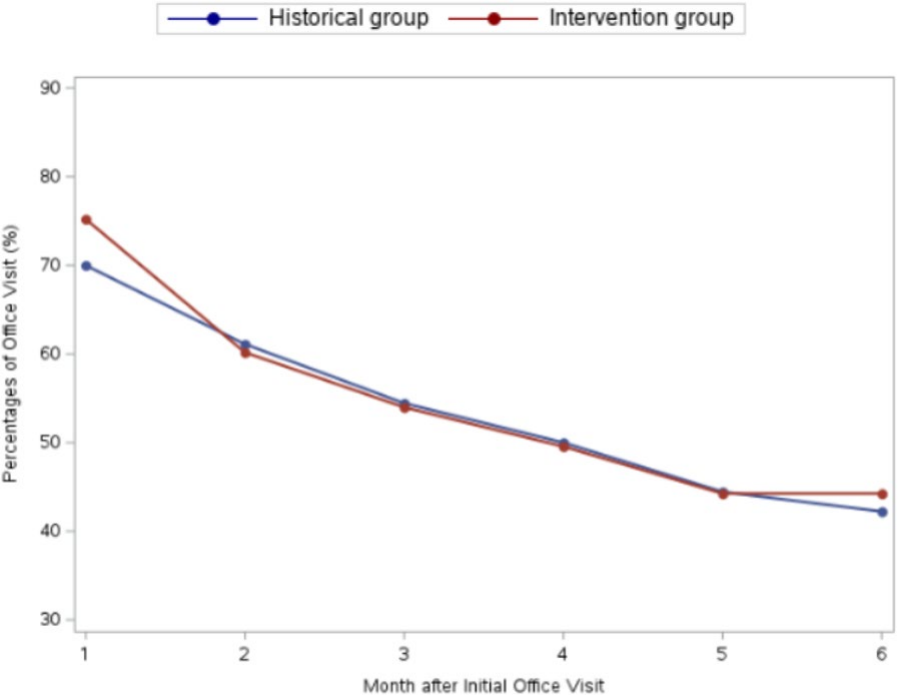
Percentage of patients with an office/telemedicine visit for MOUD by month in intervention and historical control groups

Figures 1, 2 show monthly percentages by group of MOUD prescriptions and office/telemedicine visits. Table 3 displays descriptive rates of retention in care at 3 and 6 months without excluding patients who had a care gap.

**Table 3:** Descriptive outcomes^b^

Outcome	historical control group	intervention group
N	90	113
3-month treatment retention^c^	54 (60.0%)	72 (63.7%)
6-month treatment retention^c^	42 (46.7%)	66 (58.4%)
6-month mortality	0 (0.0%)	^a^(0.8–8.8%)
6-month unintended opioid overdose	^a^(1.1–11.1%)	17 (15.0%)

^a^Cells containing a value of 1 to 10 or additional cells that allow the back calculation of those small cells were suppressed in accordance with Duke EHR cell suppression policy

^b^These are descriptive only with no formal statistical testing for group differences

^c^Retention outcomes were calculated without excluding patients who had a care gap

### Acute Care utilization

There was no difference between groups in odds of 6-month all-cause hospitalization (aOR 1.03, 95% CI 0.58–1.82). Fewer patients in the intervention group had an ED visit within 6 months (38.9% vs 44.4%), but the difference was not statistically significant (p = 0.43). The mean number of ED visits within 6 months was also lower in the intervention group (1.6 vs 1.9), but the difference was also not significant (p = 0.35) (Table 4). The adjusted odds ratio of 6-month ED utilization was 0.84 (95% CI 0.61–1.16) (Table 5).

**Table 4:** All-cause hospitalizations and ED visits within 6 months by group

Outcome	Historical control group Mean (SD), Median (IQR)	Intervention group Mean (SD), Median (IQR)	P-value
N	90	113	
All-cause hospitalizations	0.1 (0.4), 0.0 (0.0, 0.0)	0.1 (0.4), 0.0 (0.0, 0.0)	0.99
Emergency department visits	1.9 (3.9), 0.0 (0.0, 2.0)	1.6 (4.2), 0.0 (0.0, 2.0)	0.35

**Table 5:** Adjusted odds of MOUD prescription, retention in care, and acute care utilization

Outcome	Intervention vs Historical control odds ratio (95% CI)^a^	P-value
6-month MOUD prescription	4.01 (2.08, 7.71)	< 0.0001
3-month retention without care gap	1.06 (0.78, 1.44)	0.71
6-month retention without care gap	1.27 (0.93, 1.73)	0.14
6-month all-cause hospitalization	1.03 (0.58, 1.82)	0.93
6-month ED visit	0.84 (0.61, 1.16)	0.29

^a^Adjusted Odds Ratio (aOR), adjusted for age, sex, race, ethnicity, insurance status, and comorbidities

### Mortality and overdose

Descriptive rates of 6-month all-cause mortality and unintentional opioid overdose by group are shown in Table 3.

## Discussion

We found that low barrier MOUD at an FQHC engaged a patient population more likely to be uninsured or publicly insured, with these patients having four times greater odds of initiating MOUD and achieving no statistically significant difference in 3-month retention in care compared to a historical control population treated through a traditional model of care. 6-month retention was greater in the low barrier intervention group, but the association was not significant. Hospital and ED utilization were similar between groups. For treatment programs considering a low barrier approach, this study provides reassurance that access to same-day MOUD is not associated with worse outcomes compared to delayed MOUD prescribing.

Although prior studies have evaluated primary care buprenorphine outcomes and low barrier models of treatment in various settings, our study is the first to investigate the effect of low barrier MOUD initiation and a harm reduction approach with a control group. Our study is also unique in including all patients who had any treatment-seeking interaction with the program, rather than only patients who initiated MOUD, which allows for a more complete view of the care continuum. This study demonstrates the feasibility of low barrier MOUD in primary care.

Our low barrier intervention group retention rates of 61.9% at 3 months and 53.1% at 6 months are consistent with rates in a recent systematic review of buprenorphine outcomes, ranging from about 40 to 65% at 6 months. [36] In community health centers, prior studies reported similar retention with multi-visit medication initiation with both abstinence-focused [37] and harm reduction treatment philosophies [33]. Compared to our results, both an FQHC low barrier group visit model [38] and low barrier treatment within a harm reduction agency [22] reported higher retention rates (82% and 68% at 3 months and 63% and 63% at 6 months, respectively), but their analyses only included patients who attended multiple visits. Low barrier programs in non-traditional settings achieved lower retention, including among patients recently incarcerated and experiencing homelessness. [23, 25]

Patients engaged by low barrier treatment were more likely to be uninsured or publicly insured than the historical control group, likely indicating higher levels of poverty [39]. The intervention group also had a higher prevalence of diabetes, which could indicate a greater burden of chronic disease. Although statistical associations were not tested, higher mortality and overdose in the intervention group may suggest greater severity of OUD and reflect the model’s support for patients with ongoing substance use. These rates demonstrate the importance of incorporating harm reduction education and interventions into low barrier treatment [40]. Prior studies of low barrier treatment have engaged patients who have difficulty accessing traditional treatment [23–28], but less is known about how different models of MOUD comparatively engage specific populations.

Although low barrier treatment achieved higher rates of MOUD initiation, retention was not significantly improved, suggesting that additional interventions are needed beyond on-demand MOUD and a harm reduction philosophy to support retention of the higher risk population engaged. In prior studies, the effect of low barrier MOUD initiation has been mixed, with one finding no change in 30-day retention [41], and another finding greatly improved 3-month retention but with atypically low retention in the control group [42]. More research is needed to identify interventions to support retention in MOUD. Recent systematic reviews found that most psychosocial interventions, integration of MOUD with medical, psychiatric or social services, telehealth, and extended-release buprenorphine do not improve retention [43, 44]. Studies of contingency management that incentivized opioid abstinence in agonist MOUD found no improvement in retention, but contingency management in antagonist treatment that incentivized treatment adherence or attendance did improve retention, suggesting that contingency management in agonist treatment incentivizing retention should be investigated [43]. Higher buprenorphine doses are associated with better retention [45], with this effect more important early in treatment [46]. Methadone and injectable opioid agonist treatments achieve greater retention than buprenorphine, [33] highlighting the importance of making them widely available.

Although retention rates were similar, different patterns of loss to follow-up between groups may inform future interventions. In the historical control group, most patients were lost to follow-up prior to MOUD initiation after their behavioral health assessment, while in the intervention group almost all patients initiated MOUD but drop-out was high in the first months of treatment (Figs. 1, 2). After 3 months, fewer patients dropped out in the intervention group, leading to higher 6-month retention that was not statistically significant. The difference in retention between groups at 6 months was greater when not excluding patients with a care gap, as more patients in the intervention group had care gaps but returned. The trend towards higher 6-month retention may indicate that a harm reduction model is better suited to facilitate longer term retention, but more research is needed. These patterns suggest that retention efforts should be targeted to the initial months of low barrier MOUD.

This study has limitations. Patients were not randomized, so there was likely unmeasured confounding. As we used a historical control group, unmeasured factors related to treatment at the clinic, the drug supply, and the COVID-19 pandemic may have changed over time, biasing the results. For example, increasing fentanyl contamination of heroin may have led to more difficulty with buprenorphine induction during the intervention, affecting retention. The higher barrier to treatment entry in the historical control period may have selected for a more motivated patient population, and the clinic began receiving more referrals from hospitals, EDs and jails during the intervention period likely leading to unmeasured differences in groups, contributing to equivalence or non-significant findings. The study’s sample size was limited, so it is possible the lack of difference found in retention between groups was due to inadequate power. We determined baseline chronic conditions from EMR diagnoses in year prior to and including the index visit, but many patients were previously out of care, so these figures were likely an undercount. We were unable to report on homelessness, which may have been relevant to our outcomes. Although this did not limit access to MOUD, our treatment model included consistent counseling, which is not a typical or necessary feature of low barrier care.

## Conclusions

Low barrier MOUD at an FQHC engaged a higher risk patient population and achieved no statistically significant difference in retention in care compared to a traditional treatment model. Future research should determine what interventions can improve retention of patients engaged through low barrier treatment. Future studies should also include qualitative interviews with clinic providers and patients involved in low barrier MOUD. Primary care clinics and community health centers can implement low barrier treatment to make MOUD more accessible to a broader population.

## Supplementary Information

**Additional file 1: Table S1.** Baseline characteristics. **Table S2.** Measures and Outcomes

## Data Availability

The data are available from the corresponding author on request.
