# Supplementary material for: Low barrier medication for opioid use disorder at a federally qualified health center: a retrospective cohort study
Source: Addict Sci Clin Pract. 2022 Nov 5;17:60. doi: 10.1186/s13722-022-00342-1 (PMC9636799; doi:10.1186/s13722-022-00342-1)
Supplement: Supplementary file 1 — Additional file 1: Table S1. Baseline characteristics. Table S2. Measures and Outcomes [file 13722_2022_342_MOESM1_ESM.docx]

**Additional File**

**Table S1. Baseline characteristics**

| **Variable** | **Values** | **ICD-10-CM Algorithm or Definition** |
| --- | --- | --- |
| *Demographics* |  |  |
| Age | Continuous | Age as of index visit date |
| Male gender | Yes/No |  |
| Race | Categorical; white, Black/other |  |
| Ethnicity | Categorical; Hispanic vs. Non-Hispanic |  |
| Insurance status | Categorical (Uninsured, Medicaid, Private) |  |
| *Medical history* |  |  |
| Current smoker | Yes/No |  |
| Prior smoker | Yes/No |  |
| *Clinical characteristics* |  |  |
| Diabetes | Yes/No | An ICD-10-CM diagnosis code of E10.*-E13.* in any position in 1 year preceding the index visit. |
| HCV infection [Samples et al] | Yes/No | An ICD-10-CM diagnosis code of B17.10, B17.11, B18.2, B19.20, B19.21 in any position in 1 year preceding the index visit. |
| HIV infection [Cochran] | Yes/No | An ICD-10-CM diagnosis code of B20.*, B97.35, D80.6*, D80.8*, D80.9*, D83.1*, D84.8, R75.*, R97.0*, R97.1*, R97.8* in any position in 1 year preceding the index visit. |
| Staphylococcus Aureus Infection (MRSA or MSSA) | Yes/No | An ICD-10-CM diagnosis code of A41.02, A49.01, A49.02, B95.61, B95.62, Z86.14 in any position in 1 year preceding the index visit. |
| Chronic Pain [Tian] | Yes/No | An ICD-10-CM diagnosis code of G89.21, G89.22, G89.28, G89.29, G89.4 in any position in 1 year preceding the index visit. |
| Depression [Quan] | Yes/No | An ICD-10-CM diagnosis code of F31.3*-F31.5*, F31.75, F31.76, F32.*, F33.*, F34.1*, F43.10, F43.11, F43.12, F43.2*, F43.8*, F43.9*, F93.0*, F94.8* in any position in 1 year preceding the index visit. |
| Schizophrenia Spectrum and Other Psychotic Disorders | Yes/No | An ICD-10-CM diagnosis code of F20.0, F20.1, F20.2, F20.3, F20.5, F20.81, F20.89, F20.9, F21, F22, F23, F25.0, F25.1, F25.8, F25.9, F29 in any position in 1 year preceding the index visit. |
| Bipolar disorder | Yes/No | An ICD-10-CM diagnosis code of F31.0, F31.10, F31.11, F31.12, F31.13, F31.2, F31.30, F31.31, F31.32, F31.4, F31.5, F31.60, F31.61, F31.62, F31.63, F31.64, F31.70, F31.71, F31.72, F31.73, F31.74, F31.75, F31.76, F31.77, F31.78, F31.81, F31.89, F31.9, F34.0 in any position in 1 year preceding the index visit. |

**Table S2. Measures and Outcomes**

| **Outcome/Measure** | **Source** | **Definition/Assessment** |
| --- | --- | --- |
| ***Primary Outcomes*** | | |
| Any MOUD prescription within 6 months | Clarity/LCHC | Any prescription order for buprenorphine or extended-release naltrexone within 6 months (0-180 days) of index visit. |
| 3-month retention in treatment without care gap[19-20] | Clarity/LCHC | Office/telemedicine visit with a Lincoln MOUD medical or behavioral provider OR buprenorphine or extended-release naltrexone prescription in day 61-120 following the index visit in the time period (i.e. the 3^rd^ or 4^th^ months) AND no care gap within 3 months.  Care gap is defined as any 2 consecutive months without an office/telemedicine visit OR buprenorphine or extended-release naltrexone prescription. |
| 6-month retention in treatment without care gap [19-20] | Clarity/LCHC | Office/telemedicine visit with a Lincoln MOUD medical or behavioral provider OR buprenorphine or extended-release naltrexone prescription in day 151-210 following the index visit in the time period (the 6^th^ or 7^th^ months) AND no care gap within 6 months. |
| ***Secondary Outcomes*** | | |
| All-cause hospitalization | Clarity | Any inpatient hospitalization encounter within 6 months (0-180 days) from the index visit (inclusive of ED🡪IP) |
| All-cause outpatient emergency department (ED) utilization | Clarity | Any outpatient ED visit encounter (excludes ED🡪IP) within 6 months (0-180) from the index visit |
| ***Additional Descriptive Only Measures (no statistical testing by group)*** | | |
| MOUD prescription in 1^st^ month | Clarity/LCHC | Any buprenorphine or extended-release naltrexone prescription in day 0-30 following index visit. |
| MOUD prescription in 2^nd^ month | Clarity/LCHC | Any buprenorphine or extended-release naltrexone prescription in day 31-60 after index visit. |
| MOUD prescription in 3^rd^ month | Clarity/LCHC | Any buprenorphine or extended-release naltrexone prescription in day 61-90 after index visit. |
| MOUD prescription in 4^th^ month | Clarity/LCHC | Any buprenorphine or extended-release naltrexone prescription in day 91-120 after index visit. |
| MOUD prescription in 5^th^ month | Clarity/LCHC | Any buprenorphine or extended-release naltrexone prescription in day 121-150 after index visit. |
| MOUD prescription in 6^th^ month | Clarity/LCHC | Any buprenorphine or extended-release naltrexone prescription in day 151-180 after index visit. |
| MOUD prescription in 7^th^ month | Clarity/LCHC | Any buprenorphine or extended-release naltrexone prescription in day 181-210 after index visit. |
| MOUD visit in 1^st^ month | Clarity/LCHC | Any office/telemedicine visit for MOUD in day 1-30 following index visit. |
| MOUD visit in 2^nd^ month | Clarity/LCHC | Any office/telemedicine visit for MOUD in day 31-60 after index visit. |
| MOUD visit in 3^rd^ month | Clarity/LCHC | Any office/telemedicine visit for MOUD in day 61-90 after index visit. |
| MOUD visit in 4^th^ month | Clarity/LCHC | Any office/telemedicine visit for MOUD in day 91-120 after index visit. |
| MOUD visit in 5^th^ month | Clarity/LCHC | Any office/telemedicine visit for MOUD in day 121-150 after index visit. |
| MOUD visit in 6^th^ month | Clarity/LCHC | Any office/telemedicine visit for MOUD in day 151-180 after index visit. |
| MOUD visit in 7^th^ month | Clarity/LCHC | Any office/telemedicine visit for MOUD in day 181-210 after index visit |
| All-cause mortality | Clarity | Death within 6 months (0-180 days) following their index visit. |
| Unintentional opioid or heroin overdose | Clarity | An ICD-10-CM diagnosis code of T40.0X1A, T40.2X1A, T40.3X1A, T40.4X1A, T40.601A, T40.691A, T40.1X1A in a primary position within the 6 months (day 0-180) from the index visit. |
| 3-month retention in treatment | Clarity/LCHC | Office/telemedicine visit OR buprenorphine or extended-release naltrexone prescription in day 61-120 following the index visit in the time period (i.e. the 3^rd^ or 4^th^ months) |
| 6-month retention in treatment | Clarity/LCHC | Office/telemedicine visit OR buprenorphine or extended-release naltrexone prescription in day 151-210 following the index visit in the time period (the 6^th^ or 7^th^ months) |
| Care gap in 3-months | Clarity/LCHC | Any 2 consecutive months without an office/telemedicine visit OR buprenorphine or extended-release naltrexone prescription in 3 months |
| Care gap in 6-months | Clarity/LCHC | Any 2 consecutive months without an office/telemedicine visit OR buprenorphine or extended-release naltrexone prescription in 6 months |
